# Round the Hospitals

**Published:** 1917-09-22

**Authors:** 


### ROUND THE HOSPITALS.

Now that the holiday season is drawing to a close nurses are again beginning to bestir themselves to become registered members of the College of Nursing, or to make themselves more fully acquainted with the College's aims and objects, and matrons are consequently endeavouring to arrange meetings with this latter object. Miss Rundle has already promised to speak at one such meeting at Nottingham on Monday, October 8, further particulars of which we hope to announce later. Matrons who desire the attendance of a speaker to explain the College of Nursing should lose no time in writing to Miss Rundle, the Secretary, at the offices, 6 Vere Street, Cavendish Square, London, W. 1.

"Sister" writes: "In The Hospital of August 25 an instance is cited of a nurse being called upon to undergo a *viva-voce* examination for her qualifying certificate, after twelve consecutive hours on night duty. Such lack of justice and consideration is almost criminal, and one sincerely hopes that it is the exception rather than the rule in our training-schools. Unfortunately, however, it is not so generally realised as it might be that the faculties and perceptions become somewhat dulled by a spell of night duty, the continuance of which renders nurses less keenly alert than normally. In some instances even their dispositions undergo a change for the time being, due in all probability to the fact that they are living ' upside down,' as it were. It is hoped that the College will in time devise means whereby an eight-hour day can be made workable, or, at any rate, that shorter working hours should prevail; but we shall have to wait a little longer for this ideal reform. In the meantime nurses should not remain on night duty more than two or three months at a time."

Our correspondent continues: "Under existing conditions in the majority of hospitals it hardly seems possible to arrange for shorter hours of night duty, but the practice of taking the midnight meal in the ward kitchens might well be abolished. If a fairly substantial meal were provided in the dining-room, half the staff to partake of it at one time, the result would be a very pleasant break in a long, trying night. With regard to off-duty time on night duty, if it is possible to give it to the day staff during a twelve-hour working day, why cannot it be made possible for the night staff to retire in turn from the wards for an hour or two during the quiet part of the night? An arrangement such as this would, of course, necessitate a slightly larger staff on night duty, but surely this is a very minor consideration when considering the welfare of the nursing staff! As a general rule the night ' pro ' could quite well relieve the staff nurse during the early morning hours if supervised by the staff nurse of the adjoining ward, and, oh! what would nurses on night duty not give for this privilege!"

A correspondent begs us to raise the question of soup as an article of diet, more particularly for children. We have already from time to time in the articles that are appearing in The Hospital, under the title of "Food in War Time," laid stress upon the high food value of well-made thick soup, and so recently as March 24, page 306, we entered a plea for the restoration of soup to its proper place in the institution household as a valuable and inexpensive form of nourishment ready to hand. In the article on "Communal Cookery" on another page of the present issue "A Social Worker" shows that thick soups play an important part in the menus of the children's communal dinners in Hampshire, where an interesting experiment in food economy is now being made. It has been found that for young children, or for all children who are not used to it, the soup should be not too strong in meat juice. Bones, of course, should be the staple stock, and cow-heel or sheep's head makes excellent soup. Every scrap of meat left on the dishes after carving, or trimmed off by the cook before serving, should go into the stock-pot, and with the aid of cereals, such as split peas, lentils, or semolina, an appetising and nourishing dish is readily made. We shall be glad to have the views and suggestions of our readers on this important subject.

The nursing staff at La Panne Hospital, which has just been evacuated of patients and British staff owing to its bombardment by the enemy, have been frequently honoured by our Allies. Recently Sisters Garlick and Millar received the Médaille d'Honneur des Epidémies from the French Army authorities, and now Sister Edith Foulkes has been invested by the Queen of the Belgians with the Elizabeth Medal. The Queen, who takes a deep interest in the staff of this hospital, which owes its financial strength so greatly to her, has presented this special medal to the sisters who have worked at La Panne for eighteen months in the case of the Belgian and twelve months in that of the English sisters. The medal is bronze in colour, with the Queen's head on one side and that of St. Elizabeth on the other.

Colonel de Page, the medical superintendent of the hospital at La Panne, is a tireless and indefatigable worker. The necessity for the sudden evacuation of the buildings was a great grief to him, for the new hospital that he is erecting at no great distance is not yet completed. It is to be larger even than that at La Panne, which contained 1,200 beds. On the day of the evacuation of the latter there were only 200 really serious cases, and about 400 convalescents. The order for evacuation was given at 6.45 p.m., and at 10 p.m. all were gone except a few of the staff who had been wounded in the morning, and some female patients, who left the next day. All the English staff have now left La Panne, but the hope is expressed by more than one member that they may return to work at the new Hospital when it is erected.

In consequence of a breakdown in health, Miss Gwyn, lady superintendent of the Kent and Canterbury Nurses' Institute, has resigned office. Miss Gwyn's experience in the nursing profession has been long and varied, for she entered the Royal Hants County Hospital, Winchester, as a probationer in 1875. She was later promoted to be sister, and subsequently lady superintendent there, and has also held the post of matron of the Birmingham General Hospital, Worcester General Infirmary, Harrogate Bath Hospital, and York County Hospital. Miss Gwyn is therefore well known to a large number of nurses working all over the world, who will unite in fervent wishes for her renewed health and comfort in her well-earned retirement.

What promises to be a highly interesting matinée is announced to be held on October 30 at the Palace Theatre, which has been kindly lent by Mr. Alfred Butt for this purpose. It is to consist of a series of tableaux representing pictures of angels of many schools of painting, and the copy in the living material is to be so exact as to render it simple to recognise the artists' methods in each tableau. The matinée is being organised by the British Women's Hospital, and Miss Gertrude Kingston is arranging the tableaux. The music will be a great feature, and the organisers are fortunate in having secured the co-operation of Dr. R. R. Terry, of Westminster Cathedral, who is a well-known authority on early Church music. The programme of music will be rendered by a select choir, organised by Dr. Terry, which will include tenors and basses from Westminster Cathedral.

An insight into the life of Indian diplomaed nurses in Calcutta is afforded by a report of the Calcutta Corporation, which had to consider an appeal from Mrs. Sandys for a grant to the St. Luke's Hostel for Nurses. This is an institution opened in 1915, and is under the supervision of the Church Missionary Society. The nurses are available for taking cases in the city at a charge of 3 Rs. or 4 Rs. a day for ordinary, and 10 Rs. for infectious cases. The application stated that twenty-three nurses had passed through the hostel. Of the present number six were living in the hostel and three were non-resident members of the staff, and did not help at all with the expenses, the six resident nurses paid 15 Rs. per mensem, of which 8 Rs. were paid out for their food, 7 Rs. went towards the expenses, the matron's salary and food, and the rent of the flat. The total monthly expenses were 65 Rs. The income with only six resident nurses was 42 Rs., leaving a deficit of 23 Rs. per mensem. Even if there were three other resident nurses they would still not quite pay their way. There were other difficulties. A nurse's living is precarious, and when out of work they cannot always pay at once, but only when they get a case; this made the institution's income uncertain, yet the monthly expenses were regular. The hostel had protected these nurses after they left the hospital, giving them work and shelter, and had for the most part found posts for them. In the event of their leaving their posts, the hostel would again give them shelter. The nurses working with the institution had, except in one instance, proved steady in their behaviour and had received a good report from the doctors. The hostel had done and will do good work, but to carry it on help was needed, and the Corporation was asked to give an annual grant of 500 Rs. This would enable the hostel to be carried on and, if necessary, enlarged as the need grows.

The object of the institution was to raise the tone of the nurses, so that the whole profession might be uplifted. St. Luke's Hostel was inspected by the medical officer, who presented a report to the Corporation; he said that the work done by the nurses, and the appointments gained on leaving the institution, made a very satisfactory record, and spoke well for the class of nurse admitted into the hostel. The nurses were comfortably housed in the upper flat of a two-storeyed masonry building. At his inspection he was favourably impressed with the nurses in residence and with the hostel generally, but he was of opinion that a superior type of matron was an absolute necessity, if proper discipline and control were to be maintained. The Corporation decided to make an annual grant of 500 Rs., subject to the condition that a capable matron was placed in charge of the institution and that accommodation for a Corporation midwife be provided in it free.

The excellent lecture delivered by Miss Vera Matheson, B.A., Secretary to the Irish Board of the College of Nursing, to the nurses of Belfast (as reported fully in The Hospital for August 4), is being published as a pamphlet for general circulation by direction of the Hon. Sir Arthur Stanley, G.B.E., M.P. Matrons and sisters are invited to write to the Secretary of the College of Nursing, Ltd., 6 Vere Street, Cavendish Square, W. 1, stating the number of copies they would like to have. The pamphlet is entitled "A Nurse's Talk to Nurses."

☛ It is our invariable practice to pay for all items of news which we may publish. The minimum payment is 5s. Paragraphs of about twenty lines in length—that is to say, of 150 words or less—are preferred. In this section during the war we propose to give 10s. to the correspondent who may send us in any week the best incident connected with hospital life and work. In this way we hope to afford to all practical aid and encouragement; while those who reciprocate will be able to render us invaluable service. Contributions should be addressed to The Editor, The Hospital, 28 and 29 Southampton Street, Strand, London, W.C. 2, and, to be in time for the current week's issue, should reach him by the first post on Mondays.

For "Nursing Affairs in Ireland," see p 512; Matrons' and Sisters' Appointments, p. 518.

